# Ascomycota Members Dominate Fungal Communities during Straw Residue Decomposition in Arable Soil

**DOI:** 10.1371/journal.pone.0066146

**Published:** 2013-06-20

**Authors:** Anzhou Ma, Xuliang Zhuang, Junmei Wu, Mengmeng Cui, Di Lv, Chunzhao Liu, Guoqiang Zhuang

**Affiliations:** 1 Research Center for Eco-Environmental Sciences, Chinese Academy of Sciences, Beijing, P.R. China; 2 Insitute of Microbiology, Chinese Academy of Sciences, Beijing, P.R. China; 3 National Key Laboratory of Biochemical Engineering, Institute of Process Engineering, Chinese Academy of Sciences, Beijing, P.R. China; Wageningen University, The Netherlands

## Abstract

This study investigated the development of fungal community composition in arable soil during the degradation of straw residue. We explored the short-term responses of the fungal community over 28 days of decomposition in soil using culture-independent polymerase chain reaction in combination with a clone library and denaturing gradient gel electrophoresis (DGGE). Fungal cellobiohydrolase I (*cbhI*) genes in the soil were also characterized, and their diversity suggested the existence of a different cellulose decomposer. The DGGE profiles based on fungal internal transcribed spacer analysis showed different successions of fungal populations during residue decomposition. Members of *Lecythophora* and *Sordariales* were dominant in the early succession, while *Hypocrea* and *Engyodontium* were better adapted in the late succession. The succession of fungal communities might be related to changes of residue quality during decomposition. Collectively, sequences assigned to Ascomycota members were dominant at different stages of the fungal succession during decomposition, revealing that they were key drivers responsible for residue degradation in the arable soil tested.

## Introduction

Large quantities of lignocellulosic wastes consisting mainly of industrial and agricultural residues are generated worldwide every year [1]. As it is the most abundant renewable resource on the earth, diverse methods for the effective utilization of ligninocellulose have been proposed [1], [2]. In sustainable agricultural treatments, crop detritus is incorporated directly into fields, which can improve soil physicochemical properties, increase nutrient ability, and eliminate the harmful influence of agriculture waste to environments [3], [4]. The decomposition of crop residues by soil microbial communities is a key step to release inorganic nutrients from plant residues as well as transform this material into soil organic matter. It is essential to elucidate the microbial communities associated with straw residue decomposition in arable soil and understand the decomposition processes.

A broad taxonomic range of microorganisms contributes to the degradation of plant residue in soil, and microbial activities are responsible for most of the turnover of cellulose for the recalcitrant structure of this biopolymer [5]. Fungi are capable of producing a wide range of extracellular enzymes, which make them effective in attacking the intermolecular bonds of the cellulose biopolymer [1]. In fact, soil fungal communities are essential to litter degradation in forest ecosystems, and they are considered the key decomposers [6]–[8]. Using a cultivation-based isolation method, Osono and colleagues suggested that the succession of decomposer fungal communities is required to complete the litter degradation process [9], [10]. Recently, the use of molecular approaches for microbial community analysis provides a more comprehensive investigation of the complex litter decomposition process mediated by various fungal taxa [6]. Compared with forest ecosystems, lignin content in arable soil is relatively low in the chemical constituents of the crop residue input. The differences in the intrinsic quality of the substrate and the surrounding environment could have an influence on the fungal community involved in decomposition process [11]–[15]. Previous studies of fungal communities during litter decomposition have focused on activities of enzyme, microbial biomass, and phylogenetic groups in the soil based on laboratory and natural ecosystem experiments [16]–[18]. Some studies have found that members of Ascomycota and Basidiomycota represent the main soil fungal decomposers [19], [20], while others have found that members of Basidiomycota are important and are more able to degrade lignocellulose organic matter [21], [22]. Results of the general community characteristics have been mixed in early studies, and current knowledge regarding the active and functional fungal communities involved in decomposition is limited.

The application of functional genes can aid in the detection of some functional fungal groups. To this end, Edwards and colleagues recently used the cellubiohydrolase-encoding *cbhI* gene as a functional biomarker to analyze cellulolytic fungal populations, advancing our understanding of microorganisms involved in cellulose degradation [23]. Therefore, the identification of the functional *cbhI* genes of soil together with the analysis of phylogenetic information of fungal communities during straw residue decomposition is useful to gain underlying insights. The main objective of the present study was to investigate the diversity and dynamics of the fungal community throughout the degradation process. To characterize the succession of fungal decomposers, we studied short-term (28 days) responses of the fungal community via constructed microcosms using arable soil and rice straw residue. Using DNA-based denaturing gradient gel electrophoresis (DGGE) methods, we monitored the fungal community changes at different stages during the incubation. We also evaluated the metabolic activity of the fungi community in the soil we sampled through the measurement using Biolog microplates and detection of the cellobiohydrolase gene biomarker.

## Materials and Methods

### Soil and Preparation of Microcosm

The soil used for the microcosm incubation was taken from a field located in Taoyuan State Key Experimental Station for Ecological Agriculture, Hunan Province. Since 1990, a long-term experiment involving the input of residues into the soil as one organic management practice has been maintained in this field. No specific permits were required for the described field studies. The location is not privately owned, and the field studies did not involve endangered or protected species. Taoyuan State Key Experimental Station for Ecological Agriculture is one of the sites of the Chinese Ecosystem Research Network (CERN), which was implemented by the Chinese Academy of Sciences (CAS) to study environmental issues concerning China. In addition, studies of the C and N biocycle and soil science take place at Taoyuan State Key Experimental Station for Ecological Agriculture. These study fields can be used by the institutes of the CAS freely for research, and all data are shared and can be published. Our institute, Research Center for Eco-Environmental Sciences, is affiliated with CAS; thus, we can perform scientific research in these fields without specific permits. The soil samples were collected in November 2008 at a depth of 0–15 cm. Several small soil cores (approximately 5 cm in diameter) were collected randomly from each plot and mixed together as a representative sample. Each sample was placed in a sterile plastic bag, sealed, and placed on ice while transported to the laboratory. The soil was air-dried in a glass house and then filtered through 2-mm mesh to eliminate plant debris. The characteristics of the soil were as follows: pH, 4.9; total organic C, 23.2 g kg^−1^; and total N, 2.3 g kg^−1^.

The soil samples were moistened with sterile deionized water and preincubated at 20°C for two weeks in the dark to reach a biological steady state. The microcosms consisted of 100 g of soil in 500-ml glass universal bottles with screw caps. The cellulose decomposition was investigated by adding small pieces of straw residue (2 g kg^−1^ soil dry weight) to the soil. The microcosms were weighed regularly during the incubation, and water lost through evaporation was replaced through the aseptic addition of sterile deionized water. Soil amended with straw was incubated with duplicate microcosms at 20°C in the dark with weekly aeration. As controls, soil microcosms were incubated without the application of the straw. Subsamples of 10 g soil were sampled from the each treatment group on days 0, 7, 14, and 28, representing different stages of decomposition. The soil was preserved at −20°C in 50-ml Falcon tubes until used. Analysis was carried out on samples from two separate microcosms of the same treatment.

### Microbial Activity

The metabolic potential of the soil fungi communities was estimated using the Biolog™ FF plate (Biolog Inc., Hayward, CA, USA) according to the following protocol [24]: 10 g of fresh soil were suspended in an NaCl solution (0.8%) and shaken for 20 min. The sample suspensions were allowed to settle for 10 min, and then 150 µl of the sample supernatant were added to each well of the Biolog plates. Three parallel experiments were performed for the soil. The inoculated microplates were incubated at 25°C in darkness, and the optical density [25] was determined using a Biolog microplate reader for each plate at 12-h intervals over a period of 168 h at 590 nm (metabolic activity) and 750 nm (mycelial growth). The data were exported into an Excel spreadsheet for analysis. The average well color developments (AWCD) of the different replicates were calculated, where AWCD equals the sum of the difference between the OD of the blank well (water) and substrate wells divided by 95 (the number of substrate wells in the FF plates). The well color developments and growth curves were also analyzed based on the data collected from the individual cellobiose carbon source wells of the microplate [24].

### DNA Extraction and Construction of *cbhI* Gene Clone Library

The soil DNA was extracted using the FastDNA® SPIN Kit for soil (Bio 101 Inc., USA) following the manufacturer’s instructions. The DNA was eluted with 50 µl of the solution buffer supplied with the kit and visualized by electrophoresis in a 1% agarose gel. The amount of DNA was quantified using a NanoDrop ND-3300 fluorospectrometer (Thermo Fisher Scientific, Wilmington, DE). The DNA was stored at −20°C until use.

Purified total genomic DNA (∼10 ng) was used as a template for the amplification of the cellobiohydrolase I (*cbhI*) gene. Polymerase chain reaction (PCR) was used to amplify the *cbhI* gene using fungcbhIF and fungcbhIR primers according to Edwards and colleagues [23]. PCR amplification was performed in an Eppendorf mastercycler gradient PCR machine using ExTaq DNA polymerase (Takara Biotechnology (Dalian) Co., Ltd., Japan). After purification using EZNA Cycle-Pure Kits (Omega Bio-tek Inc., Doraville, GA, USA), the ampicons were cloned into *Escherichia coli* JM109 using the p-GEMT easy vector (Promega, Madison, WI) in accordance with the manufacturer’s instructions. Blue-white screening was used, and white colonies were picked at random. Colony PCR was then performed with pGEM-T primers T7F and SP6R. Clones that had yielded PCR products of the correct sizes, as determined by gel electrophoresis, were selected, and the inserts were sequenced. Sequencing of positive clones was performed with the standard primer T7F on an ABI PRISM 3730 sequencer (Applied Biosystems).

### PCR and DGGE Analysis of Fungal ITS

Amplifications of the fungal internal transcribed spacer (ITS) rRNA regions were performed using the primer sets ITS1F/ITS4 and ITS1F-GC/ITS2 using nested PCR [26]. A GC clamp (5′-CGC CCG CCG CGC GCGGCG GGC GGG GCG GGG GCA CGG GGG G-3′) was added to the 5′ end of the ITS1-F primer to prevent complete dissociation of the DNA strands. The reaction was performed in a 50-µl volume that contained approximately 10 ng of DNA, ExTaq buffer, 0.2 µM of dNTPs, 0.2 µM of each primer, and 2 units of ExTaq DNA polymerase. The amplification protocol consisted of an initial denaturation at 95°C for 5 min; followed by 35 cycles of 94°C for 1 min, 55°C for 1 min, and 72°C for 1 min; and a final elongation at 72°C for 5 min. The first round of PCR products was purified using the EZNA Cycle-Pure Kit according to the manufacturer’s instructions. A nested PCR was conducted using the purified products as the template and the primers ITS2 and ITS1F-GC using the conditions and parameters described above. All of the reaction mixtures lacking template DNA were performed as negative controls in parallel.

DGGE was carried out using a D-Code universal mutation detection system (Bio-Rad Laboratories) according to the instruction manual and 8% (w/v) polyacrylamide [acrylamide-bisacrylamide (37.5∶1)] gels containing denaturing gradients of 20–50% (100% denaturant containing 7 M urea and 40% formamide) for the separation of the PCR products. The gel was run for 17 h at 60 V at 60°C. The gels were stained in 1X TAE buffer containing 1 µg ml^−1^ of ethidium bromide, and the results were visualized under UV light. The dominant bands in the DGGE gels were excised, and the acrylamide slices were crushed and resuspended overnight at 4°C in 50 µl of sterile water to elute the DNA [27]. The recovered DNA fragments were amplified using the primers ITS1F and ITS2. The resulting PCR products were cloned into *E. coli*, and positive clones were sequenced using the same protocols mentioned above.

DNA band positions and intensities were detected using Quantity One software (Bio-Rad Laboratories). Cluster analysis was performed by the unweighted pair group method using arithmetic averages (UPGMA). The Shannon-Weaver index of general diversity (H´),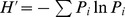, was calculated from the DGGE profiles, where *P_i_* is the relative abundance of species *i* within a community. The relative band intensity within a profile was calculated by dividing the intensity of an individual band by the total bands intensity (sum of the intensities of all bands in the lane) to minimize the variation in the quantity of loaded PCR product.

### Sequence Analysis

Raw sequence data was assembled and checked with DNAStar (Madison, WI, USA). NetGene2 was used to detect possible intron splice sites [28]. The sequences were compared with the GenBank database sequences using BLASTN and BLASTP for ITS and amino acid sequences deduced from the *cbhI* DNA sequences, respectively, and the highest matched sequences were obtained from the GenBank database (http://www.ncbi.nlm.nih.gov/BLAST/). The ITS sequences were screened for putative chimeras, as proposed by Nilsson and colleagues [29]. The final sequences were then aligned with the sequences downloaded from GenBank using ClustalW, and manual adjustments were made to the alignment where necessary. The neighbor-joining trees were constructed using MEGA version 4.0 [Molecular Evolutionary Genetics Analysis (http://megasoftware.net)] with 1000 bootstrap replicates [30].

### Nucleotide Sequence Accession Numbers

The *cbhI* gene sequences of the clone library reported here were deposited in GenBank under the accession numbers JX560501 to JX560509. The sequences of the DGGE bands were submitted to GenBank under the accession numbers JX560485 to JX560500.

## Results and Discussion

### Metabolic Activity of Fungal Community

**Figure 1:**
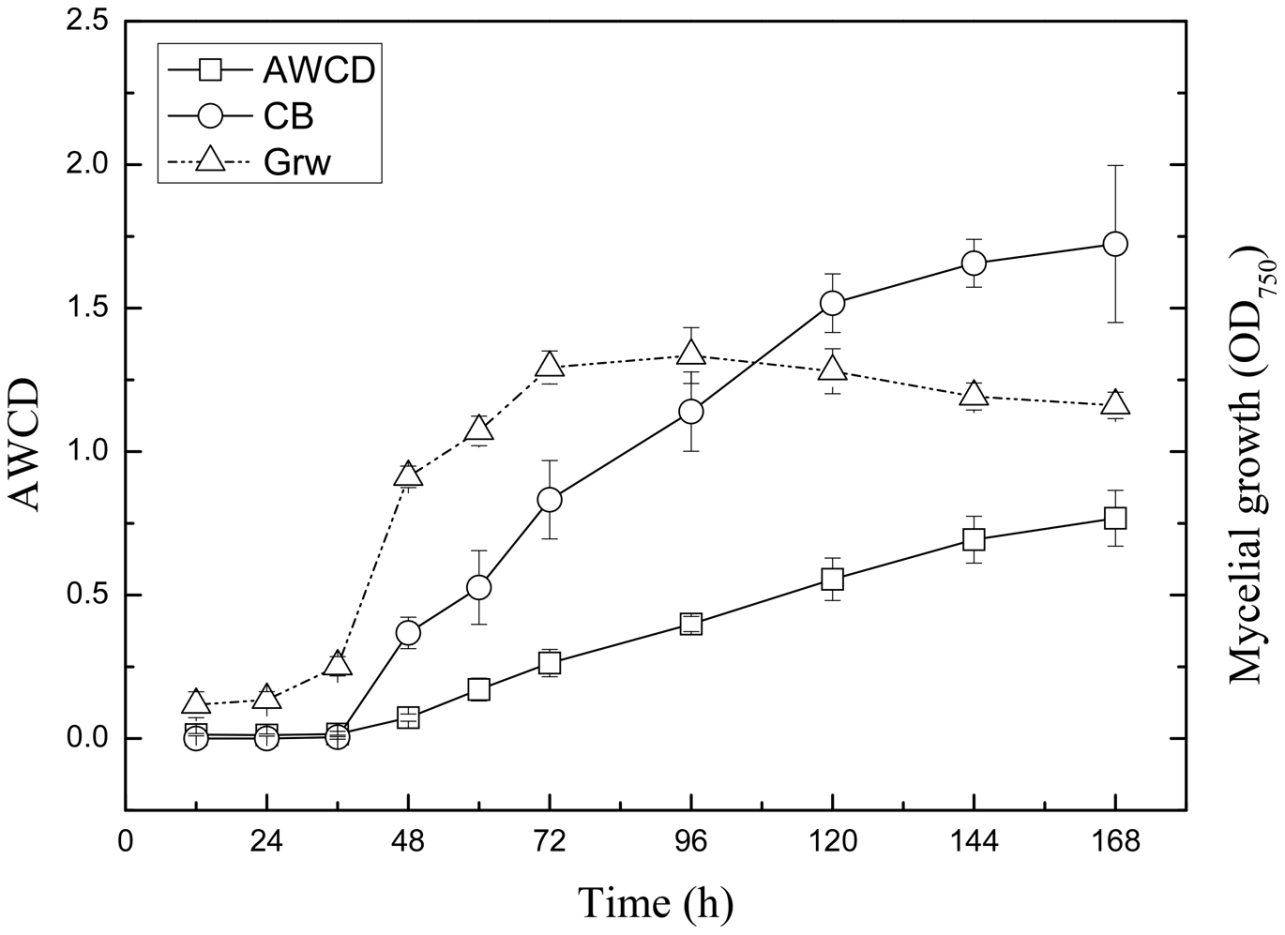
Curve results from the Biolog™ FF plate. Average well color development (AWCD) with time (squares). Growth curve (triangles) and microbial activity (circles) of soil fungi on the individual cellobiose carbon sources used in this experiment. The values represent the means and were calculated from three parallel experiments.

Fungi are known to secrete hydrolytic enzymes involved in biopolymer degradation, such as cellobiohydrolase and glucosidase. Using these enzymes, fungi can hydrolyze cellulolytic biomass [1]. To assess the heterotrophic soil fungal communities, we applied Biolog FF carbon-source-utilization-based optical density data analysis during an incubation of 168 h (Fig. 1). The average well color development values increased slowly during the incubation. Compared with AWCD, the Biolog values, based on the sole cellobiose carbon source, exhibited a significant increase, indicating that the soil fungi have a higher metabolic activity on cellobiose. Moreover, the growth of the soil fungi using cellobiose carbon is also observed in the growth curve (Fig. 1). The potential cellobiose carbon source utilization provides an independent estimate of the existence of β-glucosidase.

**Figure 2:**
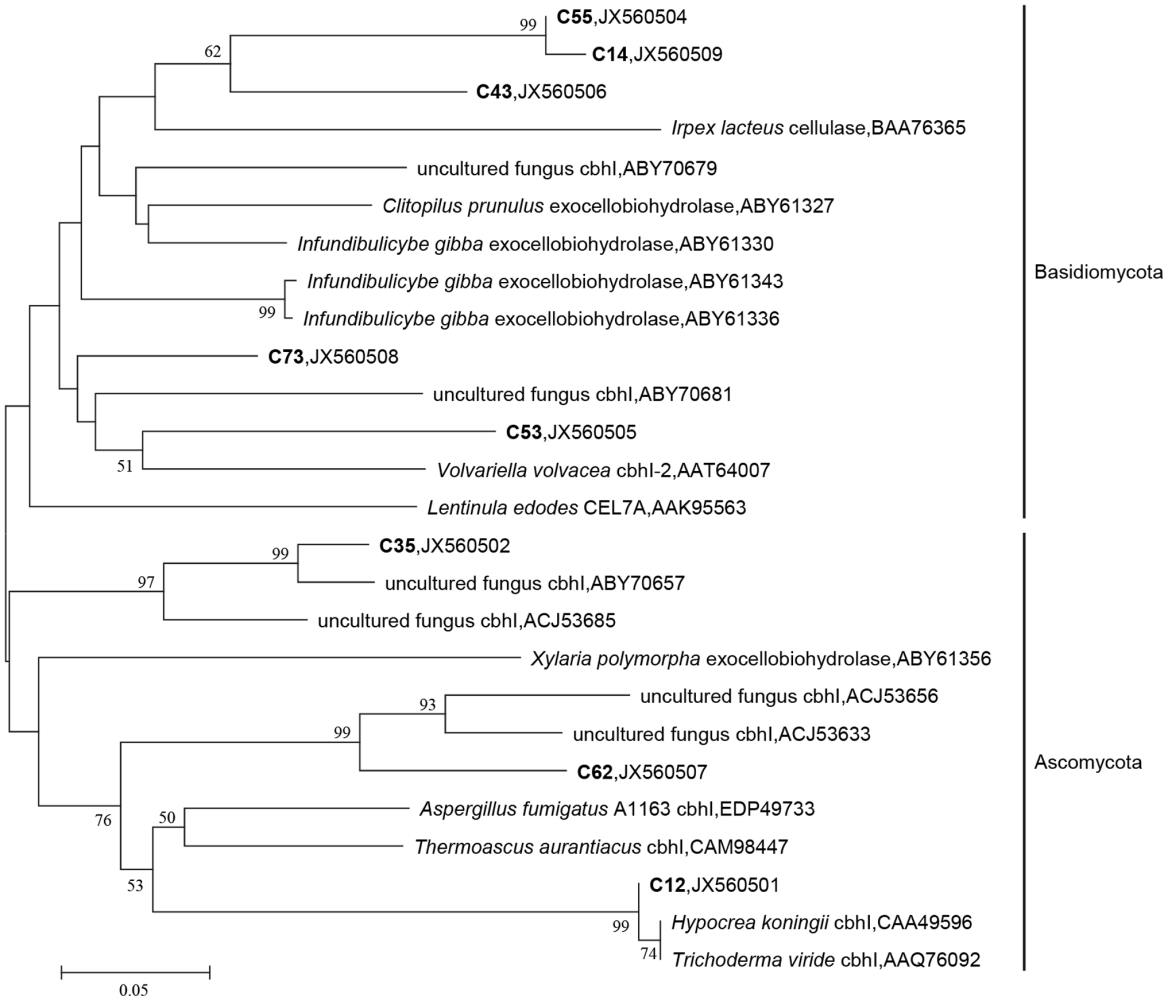
Phylogenetic relationship of the amino acid sequences derived from arable soil cellobiohydrolase I (*cbhI*) sequences. All of the sequences, except for those from this study, were obtained from GenBank. Reference sequences are shown in Roman type, and the sequences generated in this study are in boldface. The numbers on the branches refer to the bootstrap values of 1000, and bootstrap frequencies of >50% are shown.

CBHI cellobiohydrolases are key extracellular cellulase enzymes mediating cellulose decomposition to release cellobiose [31]. To characterize the *cbhI* functional gene diversity of the fungal community, we cloned and sequenced PCR products from the soil. Sequences ranging from 512–585 bp were obtained from the soil library (Table 1). Possible intron splice site analysis revealed that the majority of the sequences contained a single intron, except for C12. The translation of putative amino acid sequences ranging in length from 170–180 residues and all of the putative amino acid sequences resulted in BLASTP matches (73–99% identity) to known fungi CBHI sequences (Table 1). Pairwise similarity of the putative protein sequence ranged from 54.2–99.4%. Only C12 matched best to the known fungi *Hypocrea koningii* CBHI sequence (99%). Given that few known *cbhI* genes are deposited in public databases, it was not surprising that some of these environmental *cbhI* genotypes were not annotated to known species. The taxonomic affinity of the environmental clones were displayed by phylogenetic analysis (Fig. 2). The deduced CBHI protein fragments recovered from the soil libraries were clustered in the Ascomycota and Basidiomytoca group members (Fig. 2). In combination, the metabolic activity of β-glucosidase and the diversity of the *cbhI* functional gene reveal the cellulose degradation potential of the fungi community in arable soil.

**Table 1:** Cellobiohydrolase I gene fragments recovered from the arable soil and the putative CBHI protein.

Clone	Size(bp)	Intron 5′ splice site (size [bp])	Protein size(amino acids)	BLASTP (% identity)
C12	512	None	170	*Hypocrea koningii* 1,4-beta-cellobiosidase (99)
C35	585	424(67)	172	*Infundibulicybe gibba* exocellobiohydrolase (81)
C36	585	424(67)	172	*Infundibulicybe gibba* exocellobiohydrolase (81)
C55	561	417(49)	170	*Infundibulicybe gibba* exocellobiohydrolase (78)
C53	582	421(38)	180	*Volvariella volvacea* cellobiohydrolase I-II (75)
C43	561	418(49)	170	*Clitopilus prunulus* exocellobiohydrolase (81)
C62	566	421(50)	171	*Thermoascus aurantiacus* 1,4-beta-cellobiosidase (73)
C73	571	418(58)	170	*Volvariella volvacea* cellobiohydrolase I-II (84)
C14	562	418(49)	170	*Infundibulicybe gibba* exocellobiohydrolase (77)

### Fungal Community Dynamics during Decomposition

**Figure 3:**
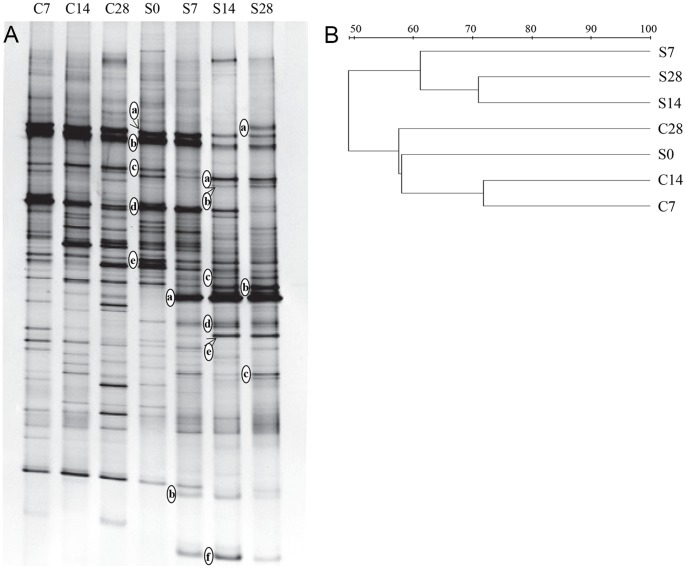
Fingerprints of the soil fungal communities from the different microcosms. (A) Denaturing gradient gel electrophoresis (DGGE) image. (B) UPGMA dendrograms based on the DGGE profiles. The lane numbers correspond to sampling points from the microcosms, and C and S represent the control and straw residue treatment microcosms, respectively. The bands marked with letters to the left of the lane were excised and sequenced.

To analyze the dynamics of the fungal community during cellulose residue decomposition, DGGE was performed on fungal ITS fragments derived from the microcosm at 7, 14, and 28 days for both treatments and on the initial soil (day 0) (Fig. 3). Although the duplication is insufficient for proper statistical assessment, reproducible profiles were obtained from both replicates for all soil treatments (Text S1). After 7 days, the DGGE profile generated from the DNA of the straw residue microcosm differed from that obtained from the DNA of the control microcosm with the appearance of new bands (S7a, S7b, and S14f), and a slight increase in the relative intensity of the existing band S0e was observed. Novel bands (S14a, S14c, S14d, S14e, S14f, and S7b) of the cellulose microcosm appeared, as compared with the DGGE profile of the control microcosm sampled at day 14. At day 28, new bands (S28a, S28c, S14a, S14e, S14f, and S7b) of the straw treatment microcosm appeared compared with the DGGE pattern of the control microcosm. In contrast, band S0c disappeared, and a decrease in the relative intensity was observed in bands S0a, S0b, and S0d. The specific band S7a of the cellulose microcosm always appeared throughout the incubation period; however, it was nearly undetectable in the DGGE profile of the control microcosm. Almost all of the bands that newly appeared in the treatment microcosms at different stages belonged to the Ascomycota based on the phylogenetic analysis (Table 2). Detailed analysis of the fungal phylogeny revealed that they were assigned to *Ustilaginoidea*, *Lecythophora*, *Engyodontium*, and *Hypocrea*. Their potential role during decomposition is discussed below. Changes in the DGGE patterns of the fungal ITS were also observed in the control treatment, which might be due to the utilization of the soil basal organic matter.

**Table 2:** Closest relatives of eukaryotes with the DNA sequences of excised ITS-DGGE bands.

Band ID	Closest species match	Similarity (%)	Phylogenetic affiliation
S7a	Uncultured soil fungus clone 53–40,DQ420800	91	Eukaryote
S7b	Uncultured fungus clone Singleton 134–1201,FJ777879	89	Eukaryote
S14a	*Calluna vulgaris* root associated fungus,FM172810	94	Eukaryote
S14b	Uncultured fungus clone RFLP49,FJ528723	83	Eukaryote
S14c	Uncultured Sordariales clone NHPY79,FJ440938	91	Ascomycota
S14d	*Ustilaginoidea viren*s,AB162148	100	Ascomycota
S14e	*Engyodontium album*,AB106650	95	Ascomycota
S14f	*Lecythophora* sp. I179,GU062252	97	Ascomycota
S28a	Uncultured endophytic fungus clone 38-1-11,EF505584	80	Eukaryote
S28b	Uncultured fungus clone LMRF_85,GU078651	90	Eukaryote
S28c	*Hypocrea virens* strain DAOM,EU280090	100	Ascomycota

**Figure 4:**
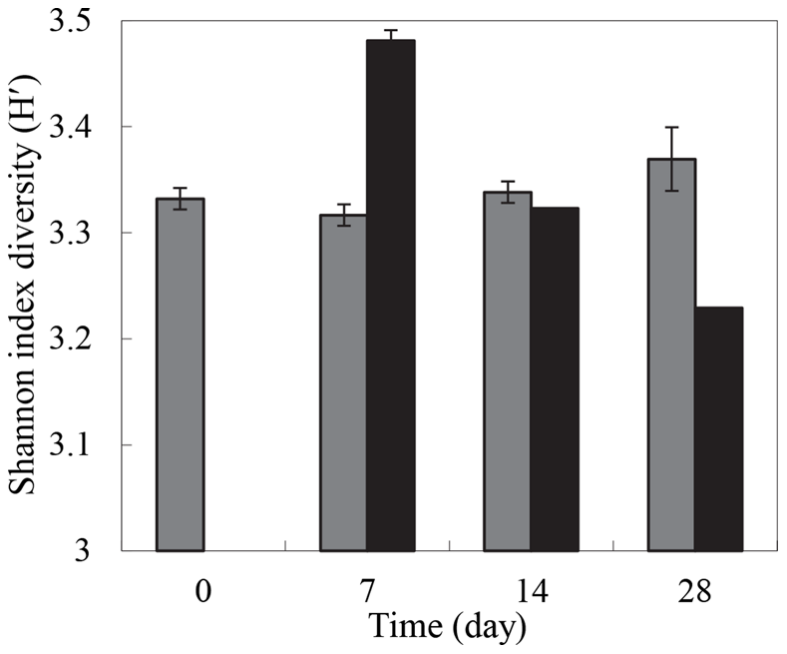
The Shannon index diversity (H´) from the DGGE profiles of the ITS fragments. The ITS fragments were amplified from the DNA of the microcosm soil with (black column) or without (gray column) the straw residue at different time points. Error bars refer to the standard error between the replicates.

**Figure 5:**
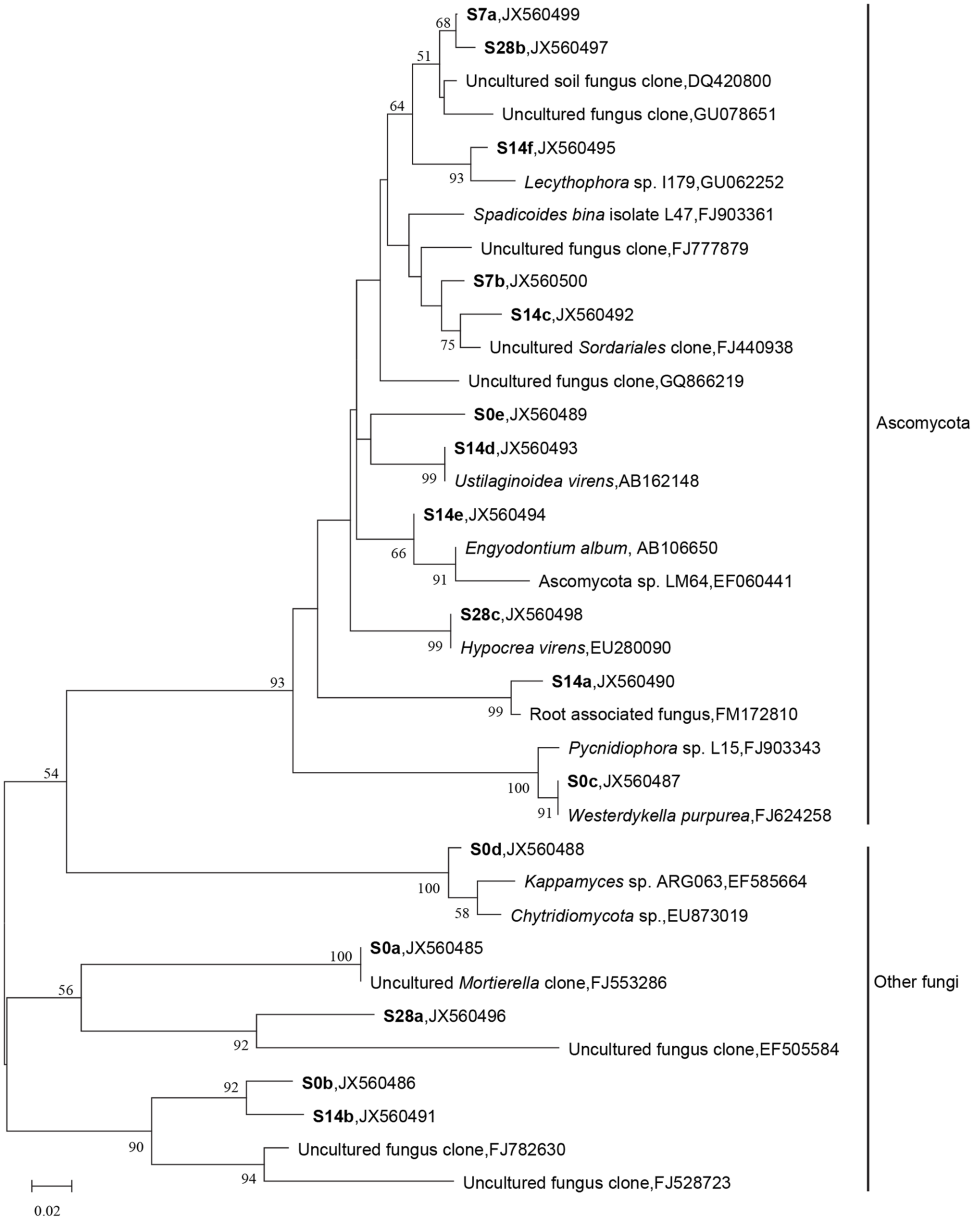
Phylogenetic relationships between the sequences of the sequenced ITS DGGE bands. The band numbers correspond to those presented in Fig. 3. The reference sequences available in GenBank are shown in Roman type, and the sequences generated in this study are in boldface. The scale bar represents the number of base substitutions per site. The bootstrap values are shown for the major branches, which had >50% support in a bootstrap analysis of 1000 replicates.

The DGGE patterns derived from the cellulose microcosm were different from the control microcosm at each sampling time point (Fig. 3A). The straw residue incorporation affected the structure of the fungal communities in the soil, which is consistent with the cluster analysis of the fungal ITS fingerprints. Cluster analysis revealed two major clusters: one formed by the residue microcosm and the other consisting of the control microcosm (Fig. 3B). The different clusters indicated that relatively similar fungal communities were involved in the cellulose microcosm and control microcosm. The fungal ITS fingerprints obtained from the cellulose residue microcosm exhibited specific profiles at each incubation time (from 0–28 days) (Fig. 3). This highlighted the existence of a succession of the microbial community during the degradation process, as described in recent studies on plant litter decomposition based on phospholipid fatty acid analyses [32]. The Shannon-Wiener indices for the samples with and without straw residue revealed that there was not a significantly greater biological diversity between the treated samples and the control. For the control microcosm, the average value was 3.35; for the cellulose residue treatment, the average value was 3.34. The diversity recovered in the control and enriched microcosms was higher than that reported in the soil environments [4]. The diversity remained similar, while the straw residue treatment significantly affected the microbial community structure and diversity (Fig. 4). The H´ value (3.48) was highest 7 days following the addition of the cellulose residue, suggesting an increase in the diversity of the fungal population. Interestingly, a lower diversity was observed over the time course in the enriched microcosm (Fig. 4). Similarly, a decrease in the active bacterial diversity has been observed after the incorporation of the wheat residue into the soil [33]. This was surprising, because an addition of a large amount of straw residue might be expected to increase the community diversity and dominance of specific fungal decomposers within the decomposition process. One portion of the carbon compounds of the straw residue, easily utilizable by soil microorganisms, might stimulate the growth of the soil fungi in early stages of the decomposition process [13]. These results suggested that the addition of the straw residue stimulated a great diversity of fungal populations affiliated with the *Ustilaginoidea*, *Lecythophora*, *Engyodontium*, and *Hypocrea* at early stages of decomposition process (Fig. 3 and Fig. 5). However, with the disappearance of an increasing proportion of labile components and the concomitant accumulation of remaining recalcitrant organic matter, most fungal populations with a limited ability to degrade recalcitrant compounds could not grow well. Within the decomposition process, the disappearing proportion of the labile component and the accumulation of the remaining recalcitrant components would regroup the related decomposers, and the novel niche with a more uniform environment might decrease the diversity of the microbial community [6].

### Fungal Genetic Structure during Decomposition

The DGGE profiles for the microcosm samples showed a clear succession of fungal populations during the straw residue decomposition. The representative bands were excised for further sequence analysis. The closest relatives of the characteristic DGGE bands (Fig. 3) mentioned above are listed in Table 2. The majority of the sequences had high (>90%) similarity to the fungal ITS sequences in the databases, except for two sequences (S14b and S28a) with low sequence similarities (<85%). The Ascomycota populations were the major active soil fungal decomposers (Table 2). The DGGE band sequences consistently clustered to the Ascomycota phylum in the phylogenetic analysis (Fig. 5). Interestingly, Osono and colleagues proposed that fungi from the Ascomycota phylum were dominant at early stages of the litter degradation process. Fungi from the Basidiomycete phylum are important in forest soil during the degradation of plant residue, especially in litter with high lignin content [25]. In crop residue in which lignin content is low, this might make fungi from the Ascomycota phylum the key decomposers in the agricultural soil tested.

The early successional group increased in the beginning stage followed by a decrease in the later stages. Bands S7b and S14f in the straw treatment were indicative of the early succession during the decomposition (Fig. 3). They were all affiliated with Ascomycota group, as shown in the phylogenic analysis (Fig. 5). Band S14f corresponded to *Lecythophora*, and some members of this genus are known to degrade lignocellulose and produce cellulase and xylanase, indicating that the fungi may contribute to the degradation of cellulose [34]. Band S7b was related to the uncultured *Sordariales* clone, an ectomycorhizosphere member [35], which remained a possibility for the production of cellulolytic enzymes that can break glycosidic bonds present in the plant cell wall. Moreover, *Sordariaceae* is one of the most well-known and best-studied families, as it consists of some notable taxa such as *Neurospora crassa*, which contains highly similar putative proteins to those shown to be associated with plant decomposition [36]. The characteristics of the related microorganisms suggest that these fungi might be involved in the primary assimilation of the cellulose during incubation.

The late successional community members gradually increased and remained at a relatively high level at the end of the incubation (Fig. 3). The late successional consumers (S7a, S14c, S14e, and S28c) were also members of Ascomycota group in the phylogenetic analysis (Fig. 5). S14e has a high similarity to *Engyodontium album*, which has ligninolytic activity and can degrade complex and recalcitrant aromatic organic matter [37], [38]. S28c shares 99% similarity with *Hypocrea virens* (syn. *Gliocladium virens*), which secretes cellulase and xylanase to *decompose* ligninocellulose, showing the ability to utilize straw residue [39]. S0a and S0b existed without significant changes over the time course of the incubation; therefore, they could be considered the non-succession group (Fig. 5). They affiliate with cellulolytic *Mortierellales* members and probably have the ability to degrade lignocellulose [40]. The ability to decompose cellulose aerobically is also widely recognized among fungi, especially among members of the Ascomycota and Basidiomycota groups. Although the Basidiomycota *cbhI* genes were detected in the sampled soil, it was interesting that members of Basidiomycota were rarely found in the sequence analysis of ITS bands during straw residue degradation in our study. Ascomycota and Basidiomycota represent the main classical fungal decomposers in different soils [19]; however, members of Ascomycota have a limited ability to degrade the recalcitrant lignin-containing litter material [41]. When soil was amended with straw, Ascomycota could use the easily degradable fraction of residues for fast-growing fungal populations. With decomposition progressing, the dissappearance of labile components and accumulation of more recalcitrant compounds would stimulate the related Basidiomycota decomposers [41], [42].

This study demonstrates the dynamics of fungal communities involved at different stages in residue decomposition. The fungal community succession can be divided into early succession, late succession, and non-succession. Importantly, Ascomycota fungi were dominant in the early and late succession communities, suggesting they were key drivers of arable soil decomposition process in our study. Furthermore, the distribution of soil fungi *cbhI* functional gene in Ascomycota and Basidiomycota suggested that the decomposition of the crop residue is a more complex and long-term process for the complete transformation of plant biopolymers. However, further research, for example, the use of stable isotope probing approaches *in situ*, is needed to identify the actual decomposers and their contribution during degradation.

## Supporting Information

Text S1 **Information of review comments including changes of** ***cbh*** **I gene and lignocellulose during straw decomposition.**
(DOC)
