# Supplementary material for: Ascomycota Members Dominate Fungal Communities during Straw Residue Decomposition in Arable Soil
Source: PLoS One. 2013 Jun 20;8(6):e66146. doi: 10.1371/journal.pone.0066146 (PMC3688710; doi:10.1371/journal.pone.0066146)
Supplement: Text S1 — Information of review comments including changes of cbh I gene and lignocellulose during straw decomposition. (DOC) [file pone.0066146.s001.doc]

Figure S1 Terminal-restriction fragment length polymorphism (T-RFLP) fingerprints of soil fungi *cbhI* gene over time during straw residue decomposition. The number means the time point of sampling.

Figure S2 Degradation of lignocellulose components during decomposition of straw residue in soil. Decomposition of cellulose (black squares), hemicelluloses (black circles) and lignin (black triangles). Error bars refer to the standard error between different samples.

Responses to the reviewer’s comments:

We sincerely thank the editor and reviewers for their insightful comments.

Reviewers' comments:
General remarks:
Comment: I would suggest to add the supplementary material provided with the response to reviewers to the manuscript as SM available to readers. 
Also, it seems that the professional editorial service that did a great job on the manuscript did not look at some of the additional pieces of text that the authors added during the revision.

Response: Thank you for your suggestion. We have added responses to reviewers to the supplementary materials and submitted them. In addition, the entire revised manuscript has been edited by another language editing company in the U.S.

Minor editorial comments:
Comment: l.73: Application of functional genes can aid in the detection of particular functional fungal groups. To this end, Edwards and colleagues recently used the cellubiohydrolase-encoding *cbhI* gene as a functional biomarker to analyse cellulolytic fungal populations, advancing our understanding.....
Response: We have replaced these sentences per your suggestion. (line 72 to line 76, page 3)

Comment: l.79: there seems to be something missing after underlying

Response: This sentence was revised. It now states as “the identification of the functional *cbhI* genes of soil together with the analysis of phylogenetic information of fungal communities during straw residue decomposition is useful to gain underlying insights.” (line 76 to line 78, page 3)

Comment: Experimental set-up: I would strongly advice to use at least triplicates in future experiments. Duplication is insufficient for proper statistical assessment of the observed results. The authors might want to add a sentence to the discussion indicating the limitations of the setup.

Response: Thank you for this great advice. We will use at least triplicates in the future studies per your suggestion. The technique limitation of duplication setup was discussed in the revised manuscript (line 248 to line 250, page 9). Although duplicated experiments are insufficient for proper statistical assessment, reproducible proﬁles were obtained from both replicates for all soil treatments. Therefore, we hope our results would be useful for the research community to further understand the posed questions.

Comment: l.165: The GC clamp is added to primers to prevent complete dissociation of the DNA strands, rather than to stabilize melting behavior

Response: We have modified the sentence per your suggestion. (line 166, page 6)

Comment: l.248: both replicates, rather than both templates
Also, please quantify the reproducibility e.g. by means of Pearson moment correlation coefficients of duplicates, or even better, a supplementary figure with all samples, incl. replicates, included in the cluster analysis.
Response: We thank you for these comments. The sentence was revised accordingly (line 249, page 9). To assess the experimental reproducibility, Pearson moment correlation coefficient was calculated based on the relative intensities of DGGE bands. Correlation coefficients of replicates for different treatments ranged from 0.785 to 0.938 (C7, 0.857; C14, 0.883; C28, 0.785; S7, 0.912; S14, 0.931; and S28, 0.938), suggesting that the consistency of the patterns of repeats was verified.

Comment: l.254: The sentence regarding the profiles at d28 is unclear and incomplete. Please check and rephrase

Response: Thank you for your suggestion. We reconstructed the sentence. It now reads as “At day 28, new bands (S28a, S28c, S14a, S14e, S14f, and S7b) of the straw treatment microcosm appeared compared with the DGGE pattern of the control microcosm.” (line 256 to line 257, page 9)

Comment: l.260: Almost all of the bands that newly appeared in the treatment microcosms at different stages ...
Response: We revised the sentence per your suggestion. (line 261, page 9)

Comment: l.263: Their potential role during decomposition is discussed below

Response: Revision was made accordingly.

Comment: l.296: However, with the dissappearance of an increasing proportion of labile components, and the concommitant accumulation of remaining recalcitrant organic matter, ...

Response: We revised the sentence as suggested. (line 298 to line 300, page 10)

Comment: l.341: probably, rather than properly
also, this sentence seems incomplete

Response: We revised the sentence, it now reads: “They affiliate with cellulolytic *Mortierellales* members and probably have the ability to degrade lignocellulose” (line 345, page 12)

Comment: l.348: As we know, ...

Response: Revision was made accordingly.

Comment: l.352: With decomposition progressing, the dissappearance of labile components and accumulation of more recalcitrant compounds would stimulate...

Response: We revised the sentence as suggested. (line 355 to line 356, page 12)

Comment: l.355: This study demonstrates the dynamics of...

Response: We deleted the word “that”. (line 358, page 12)

Comment: l.359: suggesting, rather than elucidating

Response: Thank you for your suggestion. We revised the phrase.

Comment: l.364: identify, rather than highlight

Response: Revision was made accordingly.
